# Refining Measures for Assessing Problematic/Addictive Digital Gaming Use in Clinical and Research Settings

**DOI:** 10.3390/bs5030372

**Published:** 2015-08-12

**Authors:** Kyle Faust, David Faust

**Affiliations:** 1 Department of Psychology, University of Rhode Island, 10 Chafee Road, Kingston, RI 02881, USA; E-Mail: faust@mail.uri.edu; 2 Alpert Medical School, Department of Psychiatry and Human Behavior, Brown University, Box G-A1, Providence, RI 02912, USA

**Keywords:** internet gaming disorder, gaming addiction, assessment, DSM-5, treatment

## Abstract

Problematic or addictive digital gaming (including all types of electronic devices) can and has had extremely adverse impacts on the lives of many individuals across the world. The understanding of this phenomenon, and the effectiveness of treatment design and monitoring, can be improved considerably by continuing refinement of assessment tools. The present article briefly overviews tools designed to measure problematic or addictive use of digital gaming, the vast majority of which are founded on the Diagnostic and Statistical Manual of Mental Disorders (DSM) criteria for other addictive disorders, such as pathological gambling. Although adapting DSM content and strategies for measuring problematic digital gaming has proven valuable, there are some potential issues with this approach. We discuss the strengths and limitations of current methods for measuring problematic or addictive gaming and provide various recommendations that might help in enhancing or supplementing existing tools, or in developing new and even more effective tools.

## 1. Introduction

The enormous expansion of digital technology has led to considerable interest in potential positive and negative consequences and their measurement. In this article, we will focus on measurement within a critical subdomain of digital technology that potentially impacts millions of individuals and has captured the interest of numerous investigators and the public as well, this being *digital gaming*. By digital gaming, we refer to any type of game that can be played on an electronic source (e.g., video games, computer games, mobile phone games, *etc.*).

We will first briefly overview measures designed to assess problematic digital game use and their underlying conceptual framework. We then present detailed suggestions that may assist in the further refinement or development of measures. Some of these suggestions also have potential application to measures designed to assess positive and negative consequences of other types of digital technology, or to appraise other behavioral addictions. For instance, our definition of digital gaming does not include problematic or addictive Internet use (aside from games played on the Internet). Internet addiction tools have also been the subject of scholarly reviews [1], and some of our recommendations will also (but not fully) apply to these measures. Our suggestions are not intended as negative commentary on existent measures, a variety of which possess multiple favorable qualities and have created a foundation for assessing key constructs and advancing the field. Rather, they are intended to offer possible avenues for enhancing the clinical and research utility of measures.

A word on terminology is in order before proceeding. Certain terms in this article refer to diagnostic categories that have been or are in general use, such as *Internet gaming disorder* (IGD), *pathological gambling* (PG), and its revised form, *gambling disorder* (GD). Other terms as used here, such as *addictive* or *problematic game use*, are not intended as references to formal diagnostic categories, but rather as descriptors or qualifiers. Given the intent and purpose of this article, we will not cover the potential pros and cons of using the label *addiction* when addressing excessive digital gaming. Thus, whether we use such a term as *problematic use* or *addiction*, we are not taking a position on this issue. At times we do prefer *problematic digital game use* (PDG) over *addiction* because the former is broader and includes types of excessive use that seemingly do not fit well with common conceptions of addiction.

## 2. Methods and Criteria for Assessing Problematic Digital Game Use

There is broad agreement that a subgroup of individuals who play digital games develop patterns of problematic use that may have serious negative consequences [2]. For example, concerns have been raised about an increased tendency to act violently [3]. Other concerns include the belief that escalation towards extreme levels of use could compromise many areas of everyday functioning, such as social or occupational activities [4]. Epidemiological studies have provided widely differing estimates of frequency [5,6], but even working with the lower range estimates such as 2% or 3% of gamers, multiplying such a percentage by the hundreds of millions of individuals who engage in digital gaming worldwide generates a large, if not massive, figure.

Concerns about digital gaming, and especially its potential for extreme or addictive use and resultant adverse impacts, have led to concentrated efforts to develop measurement tools. Many of these investigators have turned to the Diagnostic and Statistical Manual of Mental Disorders (DSM) for foundational guidance. We thus begin by examining how the conceptualization of problematic digital game use (PDG), particularly as described in the DSM, has shaped the development of most measures, and then discuss advantages and potential limitations of these and other conceptual approaches.

### 2.1. Use of DSM-IV-TR as a Foundational Tool

Most initial efforts to develop measurement tools for PDG used criteria that largely paralleled or adapted DSM-IV-TR criteria for pathological gambling or general substance dependence [7]. Examples include the Problem Videogame Playing Scale (PVP) [8], the Game Addiction Scale (GAS) [9], and the Problematic Online Game Use Scale (POGU) [10]. We will examine these criteria in some detail below. King, Haagsma, Delfabbro, Gradisar, and Griffiths [7] provided a scholarly review of such measures, and it is instructive to describe their conclusions in some detail.

King *et al.* covered 18 instruments, all of which used criteria quite similar to those contained within DSM-IV-TR’s categories for pathological gambling or general substance dependence [11]. King *et al.* concluded that most of the measures possess multiple positive qualities, such as brevity, ease of scoring, strong internal consistency, and strong convergent validity. In addition, various measures seem suited to collecting important information for a range of purposes, such as developing normative databases.

King *et al.* identified areas of concern, including inconsistent coverage of diagnostic criteria, differing cut-off scores (thereby compounding problems discerning true pathologic use or comparing rates across studies using contrasting measures), lack of a temporal dimension, and inconsistent dimensionality. For example, factor analysis yielded a single common dimension for a number of measures, which seemed to represent PDG, but two or more dimensions for other measures, such as compulsive use, withdrawal, and tolerance. The authors also provided suggestions for improving measurement, such as adding time-scales and validity checks (e.g., examining whether the gamer or the gamer’s family believe their gaming is problematic), obtaining data from expanded or more representative samples, and studying the sensitivity and specificity of various tools. In this article, we hope to add to King *et al.*’s useful suggestions.

### 2.2. Publication of DSM-5 and Changes in Diagnostic Categories and Criteria

King *et al.*’s review [7] appeared shortly before DSM-5 [12] was published and thus did not cover revisions in the manual, in particular the creation and introduction of the category, *Internet gaming disorder* (IGD), under the section, “Conditions for Further Study.” In response to this revision, some researchers directly adopted the DSM-5 criteria for IGD to assess problematic digital gaming. It might be assumed that IGD only applies to online games, but the “Subtypes” section of DSM-5 indicates that IGD “could also involve non-Internet computerized games as well, although these have been less researched” [12].

The IGD diagnostic criteria are quite similar to both the old DSM-IV-TR criteria for pathological gambling and DSM-5’s modified version of these criteria under the renamed category, *gambling disorder* (GD). Under DSM-5, the only major difference between IGD and GD comes down to a single diagnostic criterion: IGD does not include one of the diagnostic criterion for GD (“Relies on others to provide money or relieve desperate financial situations caused by gambling”), and rather uses, “Loss of interests in previous hobbies and entertainment as a result of, and with the exception of, Internet games.”

Recently, Pontes and Griffiths [13] published a brief measure called the Internet Gaming Disorder Scale. This questionnaire uses the nine DSM-5 IGD criteria in a 5-point Likert scale format. Pontes and Griffiths [13] studied a sample of 1060 gamers, and indicated that the measure, along with IGD, could provide a unified method of assessing video game addiction.

Those using DSM-5 might assume that IGD potentially encompasses a range of Internet activities, such as online gambling disorder (because online poker could debatably be considered a digital game). Hence, a key clarification is in order: DSM-5 states that IGD does not include use of the Internet for purposes other than gaming, such as recreational or social Internet use [12]. It further states that Internet gambling is not included in IGD [12].

### 2.3. Further Consideration of Diagnostic Criteria and Categories

At this point, it might seem as if these various criteria for problematic digital gaming are very similar. After all, the IGD criteria differ minimally from DSM criteria for pathological gambling or gambling disorder. Further, most alternatives, such as a well-known addiction model developed by Brown and modified by Griffiths [14], appear to overlap considerably with these other diagnostic criteria. Consequently, it might be assumed that so long as the various assessment tools cover such criteria, they are all likely to measure about the same thing. It may also seem to follow that the IGD criteria should become the new, preferred method for assessing digital gaming problems, especially because they were proposed in the latest DSM. Indeed, some researchers [13,15] have recommended that future measurement should consist of items that best reflect the nine IGD criteria.

Unfortunately, the situation is probably not that simple, as these measures are not free from some limiting or problematic features. For example, it is not clear that all pertinent criteria or constructs of PDG have been properly captured to date, and some of the criteria and constructs that apply to GD may have limited or minimal value for identifying PDG and *vice versa*. As such, it is important that we stay open to modifying existent criteria or adopting new criteria for PDG and IGD given the rapid evolution of digital technology and emerging research findings.

## 3. Improving/Refining Measures

The following sections provide recommendations that might further improve existing measures, or lead to the development of even stronger measures.

### 3.1. Need for a Specific Definition of Problem Gaming

Regardless of what the problem is called (PDG, IGD, or gaming addiction), a term needs to be established that properly includes all types of digital games. We believe that *digital gaming* achieves this goal, but numerous researchers use the term *video games* when they intend to refer to all types of digital games, whereas other researchers use this term when they are referring exclusively to video console games (which is why we have also used *video games* when citing certain researchers in this article).

Another important consideration in achieving a concrete definition of PDG is deciding what counts as digital game play. For a researcher with less background in this area, this may seem like a foolish question, but many digital gamers spend significant amounts of time *watching* digital games. Similar to professional sports viewers, some players likely spend more time watching or talking about digital games than they do playing them. These gamers may watch their friends play, or they may watch videos of game playing online, where they are often able to interact with skilled players. Skilled gamers may also spend time watching recorded videos to analyze their gameplay, or use chat programs to communicate with other players about different games. It remains unclear if research on the various 18 assessment tools that King *et al.* [7] reviewed accounted for this type of digital game use. If not, it is likely that some respondents did, and some did not, count time spent watching digital games when answering questions, as some gamers would consider watching games different than playing them. Attempting to appraise and reduce these ambiguities are worthwhile goals.

The question of what type of gaming activities to count raises additional questions. Should researchers count time gamers spend talking about digital games among their friends in a social situation as digital game use? If not, would it count as digital game use time if the gamer instead had a conversation over the Internet? Why, or in what way, should online social interaction be viewed differently than real-life social interaction? The implications of these questions are very important, particularly because researchers and clinicians might disagree on answers, and the availability of scientific data to resolve differences in viewpoint may be scant. For the time being, perhaps all of these differing modes of involvement in digital gaming should be captured in some way. It remains unclear how different the effects of *watching* or *analyzing* digital games are from playing them, but beginning to study these differences and incorporating the differences into questionnaires would likely be beneficial.

### 3.2. Adequate Coverage of Content: Accounting for Positive Effects

A factor that makes PDG an especially interesting issue is the benefits digital gaming may produce [16,17]. Examples include improvements in reaction time [18], spatial resolution and visual processing [19], working memory [20], cognitive flexibility [21], strategic problem solving [22,23], and prosocial behavior [24]. Even PDG, despite adverse impacts, may simultaneously produce these or other benefits.

Although a central reason to assess PDG is to determine if digital games are impacting a person’s life negatively, it may be a mistake to disregard benefits that could also be occurring. This is not to fault current measures for focusing on adverse impacts, which often are of central interest and concern. That said, it should be possible to create questionnaires that evaluate both the potential pros and cons of digital gaming. Such a questionnaire would likely be regarded far more positively by gamers, as many gamers (regardless of whether their digital game use is problematic) are frequently bothered by responding to questionnaires they perceive as having a strong negative bias towards gaming. Researchers have sometimes described challenges recruiting gamers to participate in studies, and the presence of positive items on questionnaires and interest in potential positive impacts might go a fair distance in increasing involvement and improving the representativeness of samples. In addition, measurement of positive and negative features could prove very helpful in longitudinal studies that examine cross-over from benign or relatively benign patterns of use to more problematic ones, or subsequent movement from problematic to less problematic use.

As a specific example, the evaluation of treatment programs might benefit from measurement that attends to not only negative impacts but also more benign and even positive impacts. Weighing both the pros and cons of gaming could also be particularly useful in developing treatment plans. If a gamer is experiencing both positive and negative effects from gaming activities, treatment could first involve reducing gaming use to more moderate levels, particularly if a gamer is unwilling to immediately quit gaming entirely. Ideally, lowering gaming time would reduce or remove some of the more negative impacts of gaming, while the positive impacts could continue. If the gamer is an extremely problematic user and is unable to moderate his or her use in this manner, setting more extreme limitations may be necessary.

Currently, a standard method of measuring the positive impact of digital gaming seems to be lacking. When assessing positive gaming impact, researchers have typically used measures that do not involve digital games. For example, in a study assessing the potential influence of both prosocial and violent video games, Saleem, Anderson, and Gentile [25] used the 25 item Prosocial Tendencies Measure to examine whether participants had more prosocial tendencies after gaming. Other researchers, such as Glass, Maddox, and Love [20], have used various neuropsychological measures prior to and after exposing participants to digital games to determine if the games led to cognitive improvements.

Based on these previous approaches, some suggestions can be provided for the development of positive impact item content and topics. These include asking gamers or respondents: (a) how often they engage in games that involve a lot of physical activity, such as *Dance Dance Revolution*; (b) if they make a financial living off of gaming, such as being a professional gamer or a professional gaming commentator; (c) how often they engage in social activity while gaming; (d) the different types of games they engage in (as some games appear to have more pros or more cons than other games); and (f) some of a gamer’s perceived benefits of gaming (which might prove useful for developing treatment plans for gamers in need of intervention). It would likely also prove informative to use a brief prosocial measure (such as the Prosocial Tendencies Measure [25]), and one or more brief cognitive measures that cover areas in which research has demonstrated improvements.

### 3.3. Accounting for Careless and Random Responding

The value of a self-report measure can be seriously compromised when respondents fail to cooperate sufficiently with procedures and engage in careless or random responding. Some respondents, for example, wish to complete questionnaires as quickly as possible, and in many situations the anonymity of research creates almost no barriers to careless or random responding. Investigations show that careless and random responding to questionnaires are more common than may be assumed, with rates sometimes running as high as 20% [26,27]. Furthermore, even a relatively small proportion of careless or random responders may have a surprisingly robust impact on research data and may cause paradoxical effects (e.g., not only impeding the detection of true relationships, but even creating artifactual associations between variables that are actually unrelated [28]).

It fortunately turns out that often just a few items can achieve a high level of accuracy in identifying random responding, and moderate to high accuracy in detecting careless responding. Such a small item set should take most respondents well under a minute to complete. In addition, random and careless response items may well retain effectiveness when applied or adapted across measures, or can easily be modified to blend into the content of questionnaires. Thus, an effective and simple method of improving current PDG assessment tools is to include a few careless or random response questions, which would allow researchers to identify and remove most of these non-cooperating individuals and hence attenuate their potentially damaging impact considerably.

### 3.4. Improved Norms and Reference Groups

It is often difficult to interpret the outcome of a measure if appropriate normative or reference groups are lacking. In this context, by *normative groups*, we are referring to members of the general population who presumably are not addicted or problem users. Alternatively, one might prefer a more stringently defined normative group that is comprised of general population members who are free of psychiatric disorder. The term *reference group* is broader than normative group and can be used to refer to any comparison group that might be informative relative to the group of interest (which in this domain is likely to be problem digital gamers).

Normative groups and reference groups often provide crucial information, such as the frequency with which characteristics used to identify individuals within a diagnostic category occur in other groups. For example, some proposed criteria for problematic digital gaming refer to types of dysfunction that are not specific to this activity (e.g., academic or occupational dysfunction) but are observed among a certain percentage of the general population and perhaps many individuals with certain clinical disorders. Relative frequency of occurrence across these various groups provides valuable guidance on the usefulness of proposed diagnostic criteria, such as whether or how successfully they distinguish effected individuals from members of the general population, or assist in differential diagnosis. For example, a characteristic that is common among problematic video gamers but rare among the general population likely has some utility, but if these same characteristics occur as often or more often among various clinical groups they may have little or no utility for differential diagnosis. Obviously, determining whether potential signs and indicators separate out individuals with PDG from those without PDG and how accurately they do so, and whether or the extent to which they help with differential diagnosis, can provide invaluable assistance to clinical and research efforts. For example, deriving effective or optimal cut-off scores requires such information.

As noted in the previous discussion of content domain and the potential advantages of adding positive items, recruiting gamers to participate in studies has posed challenges. For example, problematic or frequent users may distrust researchers and suspect a negative agenda. Given the considerable value of developing quality normative and reference group data, the effort seems worth it. There is much to be gained by expanding normative data bases, making it a clear priority in measurement design, development, and selection.

### 3.5. Studies on Sensitivity, Specificity, Positive Prediction, and Negative Prediction

Sensitivity refers to the frequency with which a disorder that is present is detected, and specificity to the accuracy with which the absence of disorder is identified. Both qualities need to be studied because there is an inevitable trade-off between the two (unless a diagnostic method is perfect). Ill-derived cut-off scores may produce very impressive results for sensitivity but abysmal results for specificity, and *vice versa*. A measure has limited or no value (and marked potential for harm) if it almost always identifies a disorder but almost always misidentifies normal individuals as abnormal, or if the reverse occurs. Such outcomes are functionally similar to discarding the measure and identifying most everyone as abnormal, or most everyone as normal.

Sensitivity and specificity also provide the basis for determining *positive predictive power* and *negative predictive power*, which adjust figures for sensitivity and specificity in accord with the base rate for disorder in the population of interest. In this context, adjusting sensitivity and specificity in relation to base rates allows one to determine how often a positive or negative result on a diagnostic indicator will identify PDG or lack of PDG correctly. Clinicians and researchers use assessment measures in conditions and settings in which base rates can vary considerably, and hence reporting not only sensitivity and specificity but also positive and negative predictive power could offer essential practical guidance for developing, evaluating, and applying PDG measures.

### 3.6. Studies Examining Risk Factors and Course

For questions pertaining to onset, course, and prognosis, there is often no replacement for longitudinal studies. Longitudinal studies are rarely easy to conduct, but these troubles are often more than offset by the value of such research [29,30], including the generation of information that may be difficult or nearly impossible to capture through cross-sectional designs. Using longitudinal studies to expand knowledge about onset and course could provide substantial assistance in advancing the understanding of causal pathways, identifying factors that foster resilience or increase risk, determining if and when preventative steps are warranted, and evaluating the need for therapeutic intervention. For example, better understanding of risk and protective factors could be especially beneficial for preventing PDG before such difficulties exert a truly detrimental impact on a person’s life. It is for these reasons we suggest that when selecting or developing questionnaires, serious consideration be given to including items that address potential risk and protective factors for PDG, such as the risk factors that Rehbein *et al.* [31] and other researchers [32] have uncovered.

A newly emerging and increasingly prevalent risk factor involves games that allow players to spend actual money while gaming to improve the game or their gaming characters [33]. It seems probable that engagement with such games overlaps with, but is distinguishable from, gambling disorder, and that the amount of money spent gaming will become a good predictor of PDG. Although these purchases may have a positive impact on a player’s sense of enjoyment or wellbeing when used in moderation [33], the purchases could quickly get out of hand for a gamer who struggles with impulse control. Those developing assessment tools may wish to examine real-life money spent for “in-game” purchases as a potential predictor (or criteria) of problematic use. However, this predictor would require critical analysis, as a gamer with substantial financial resources could spend considerably more money on in-game purchases without experiencing any significant adverse consequences in comparison to a gamer with less monetary resources.

### 3.7. Comparative Studies

Thanks to the efforts of talented researchers, various measures are now available with differing degrees of supportive validation evidence. Given the array of measures, proper selection for clinical and research uses would be greatly assisted by knowing more about how they compare to one another. For example, some PDG measures may exceed others in identifying problem users, others may be superior for treatment planning, and still others may be better suited for certain age groups. To identify the measure or measures most effective for intended applications in research and clinical settings, comparative studies are needed.

### 3.8. Measures Adjusted for Age, Language, and Cultural Factors

PDG measures designed for adults have often been used with children and adolescents without examining the need for modification. In addition, language factors and cultural differences may exert a major impact on the utility of measures and the extent of generalization across groups. Terms and phrases may have non-equivalent connotations across cultures, and translation or interpretation may inadvertently change the meaning of test items. For example, a term of endearment in one culture may reflect dislike in another culture. Cultural and linguistic considerations are particularly important in the area of digital gaming given its international reach and applicability across broad socio-demographic strata. Consequently, cross-cultural research on measures would be of great potential value. For those who may be interested, Hambleton, Merenda, and Spielberger [34] provide an excellent source on adapting measures across cultures.

### 3.9. Measurement of Time Frame, Severity, and Outcome

PDG measures that incorporate temporal dimensions would increase their value. Even one or two questions addressing when someone first engaged in digital gaming, and whether, for example, level of play has decreased, increased, or remained stable over the last year would provide some indication of duration and trajectory of usage. Inquiring about patterns of use over time cannot substitute for longitudinal studies, but it at least expands the snapshot of usage across a longer time frame. As noted previously, research that incorporates temporal patterns can assist in identifying risk and protective factors, potential causative factors, predicting course over time, and distinguishing between pathology that is partly or largely independent of engagement in digital gaming and pathology that is accelerated or caused by use.

## 4. Conclusions

Most measures used to assess PDG have incorporated or relied heavily on DSM criteria, with recent extension by some researchers to the measurement of IGD using criteria set forth in DSM-5. Although various measures developed to date have a number of positive features and one or multiple supportive studies, there are some limitations to these approaches. Fortunately, there are a number of ways measurement can be further strengthened. Some of the suggestions we provided (e.g., accounting for careless/random responding, incorporating data from longitudinal studies, *etc.*) can also be applied to improving a wide range of assessment tools. It is strongly recommended that more measures include appraisal of both the positive and negative impact of digital gaming, as this will create a more balanced picture of how these activities impact lives and should provide information helpful to treatment planning and monitoring. As digital gaming continues to become more prevalent across many different countries and cultures, so too will it become increasingly important to further refine the state of measurement and the assessment of PDG. With improved measurement, it will become far more feasible to properly assess and provide assistance to individuals who are at risk for, or are currently engaged in, problematic digital game use.
